# Presenilin 1 deficiency suppresses autophagy in human neural stem cells through reducing γ-secretase-independent ERK/CREB signaling

**DOI:** 10.1038/s41419-018-0945-7

**Published:** 2018-08-29

**Authors:** Cheong-Meng Chong, Minjing Ke, Yuan Tan, Zhijian Huang, Ke Zhang, Nana Ai, Wei Ge, Dajiang Qin, Jia-Hong Lu, Huanxing Su

**Affiliations:** 1 State Key Laboratory of Quality Research in Chinese Medicine, Institute of Chinese Medical Sciences, University of Macau, Macao, China; 2 Centre of Reproduction, Development and Aging, Faculty of Health Sciences, University of Macau, Macao, China; 3 CAS Key Laboratory of Regenerative Biology,Guangdong Provincial Key Laboratory of Stem Cell and Regenerative Medicine, South China Institute for Stem Cell Biology and Regenerative Medicine, Guangzhou Institutes of Biomedicine and Health, Chinese Academy of Sciences, Guangzhou, 510530 China

## Abstract

Autophagy impairment is commonly implicated in the pathological characteristic of Alzheimer’s disease (AD). Presenilin 1 (PS1) expression in human brain gradually decreases with age and its mutations account for the most common cases of early-onset familial Alzheimer’s disease (FAD). The dominant autophagy phenotypes occur in PS1-knockout and PS1 mutant neurons; it is still unknown whether PS1 deficiency causes serious autophagy impairment in neural stem cells (NSCs). Herein, we generated the heterozygote and homozygote of PS1 knockout in human induced pluripotent stem cells (iPSCs) via CRISPR/Cas9-based gene editing and differentiated them into human NSCs. In these human PS1-deficient NSCs, reduced autophagosome formation and downregulated expression of autophagy–lysosome pathway (ALP)-related mRNAs, as well as proteins were observed. Mechanistically, ERK/CREB inhibition and GSK3β activation had key roles in reducing TFEB expression in PS1-knockout NSCs. Pharmacological inhibition of GSK3β upregulated the expression of TFEB and ALP-related proteins in PS1-knockout NSCs, whereas this effect could be blocked by CREB inhibition. These findings demonstrate that PS1 deficiency causes autophagy suppression in human NSCs via downregulating ERK/CREB signaling.

## Introduction

Alzheimer’s disease (AD) is the most common neurodegenerative disease, which is characterized by the symptoms such as progressive loss of memory and cognitive impairment^1^. AD is classified into two forms: sporadic AD and familial AD (FAD). FAD is caused by rare autosomal-dominant gene mutations such as *PSEN1*, *PSEN2*, or *APP*. Among them, *PSEN1* mutations account for the most common cases of early-onset FAD^2^. *PSEN1* gene encodes presenilin 1 (PS1), which is expressed in most human tissues. In brain, PS1 mainly presents in the hippocampus and neocortex where they are associated with learning and memory^3–5^. However, its expression in human brains gradually decreases with age^5,6^. In addition, it is well known that PS1 mainly acts as the catalytic component of the γ-secretase complex for the intra-membranous cleavage of type 1 membrane proteins, such as amyloid-β precursor protein (APP), N-cadherin, and Notch^7–9^. The neuroprotective effects of PS1 also has been reported in various models via activating several survival signals such Akt and ERK^10–12^. Therefore, reduced PS1 function is considered to associate with neurodegeneration in AD^13,14^.

Macroautophagy (autophagy) is an intracellular self-degradative process that delivers cytoplasmic substrates to lysosomes and has a critical role in the basal turnover of organelles and proteins, as well as cell survival^15,16^. Autophagy failure is commonly implicated in the pathological characteristics of AD^17–20^. Increasing evidences support that the function of PS1 affects autophagy process^21–25^. Serious impaired autophagy phenotypes such as accumulation of autophagic vacuoles and lysosomal dysfunction are observed in PS1-knockout mouse blastocysts, PS1-knockout mouse neurons, and mice brains with reduced levels of PS1^21,22^. However, one interesting study indicates that autophagy substrates are degraded efficiently in mouse blastocyst-derived embryonic stem cells (ESCs) lack PS1^26^. Neural stem cells (NSCs) is one kind of stem cells with the characteristics of self-renewal and the ability to differentiate to all neural lineage cells. Although the dominant autophagy phenotypes occur in PS1-knockout neurons, it is still unknown whether PS1 deficiency causes serious autophagy impairment in NSCs stage.

Human induced pluripotent stem cells (iPSCs) are derived from somatic cells by reprogramming technology. They can be differentiated into any cell types found in the body. Therefore, human iPSCs hold great promise for studying the gene function in specific cell types due to their unique differentiation feature^27^. Herein, we used CRISPR/Cas9 to generate isogenic human iPSCs with heterozygous and homozygous PS1 knockout and differentiated them into NSCs for evaluating the function of PS1 in regulating autophagy. We found that PS1 deficiency reduced autophagosome formation and downregulated the autophagy–lysosome pathway (ALP)-related genes at transcriptional and translational levels in human NSCs. Our study further underscored that the ERK/CREB signaling pathway mediated by PS1 in a γ-secretase-independent manner has a critical role in suppressing autophagy in human NSCs.

## Results

### Generation of human iPSC-derived NSCs with heterozygous and homozygous PS1 knockout

Serious autophagy impairment has been observed in the blastocysts and neurons isolated from the homozygous PS1-knockout mice^21,28^. We sought to examine whether PS1 deficiency-induced autophagy defects occur in human NSCs. Thus, we first generated human iPSCs with the heterozygous PS1 knockout (PS1^+/−^) and homozygous PS1 knockout (PS1^−/−^) via using CRISPR/Cas9 (Supplementary Figure 1). Human UC-H1-iPSCs were established by reprogramming urine cells derived from a healthy donor following our previous protocol^29^. iPSCs were transfected with guide RNA (gRNA) and Cas9 plasmid for generating double-strand break in the exon 4 of *PSEN1*. Sanger sequencing showed that an about 200 bp insertion in the exon 4 of *PSEN1* resulted in a premature stop codon (Supplementary Fig. 1). The analysis of quantitative reverse transcription PCR (qRT-PCR) showed that the PS1 mRNA expression levels in heterozygous and homozygous PS1-knockout iPSCs decreased to a half and almost none, respectively, compared with PS1^+/+^ iPSCs (Supplementary Fig. 1). These human PS1-knockout iPSC lines exhibited the pluripotent biomarker OCT4 and retained normal karyotypes (Supplementary Fig. 1).

**Fig. 1:**
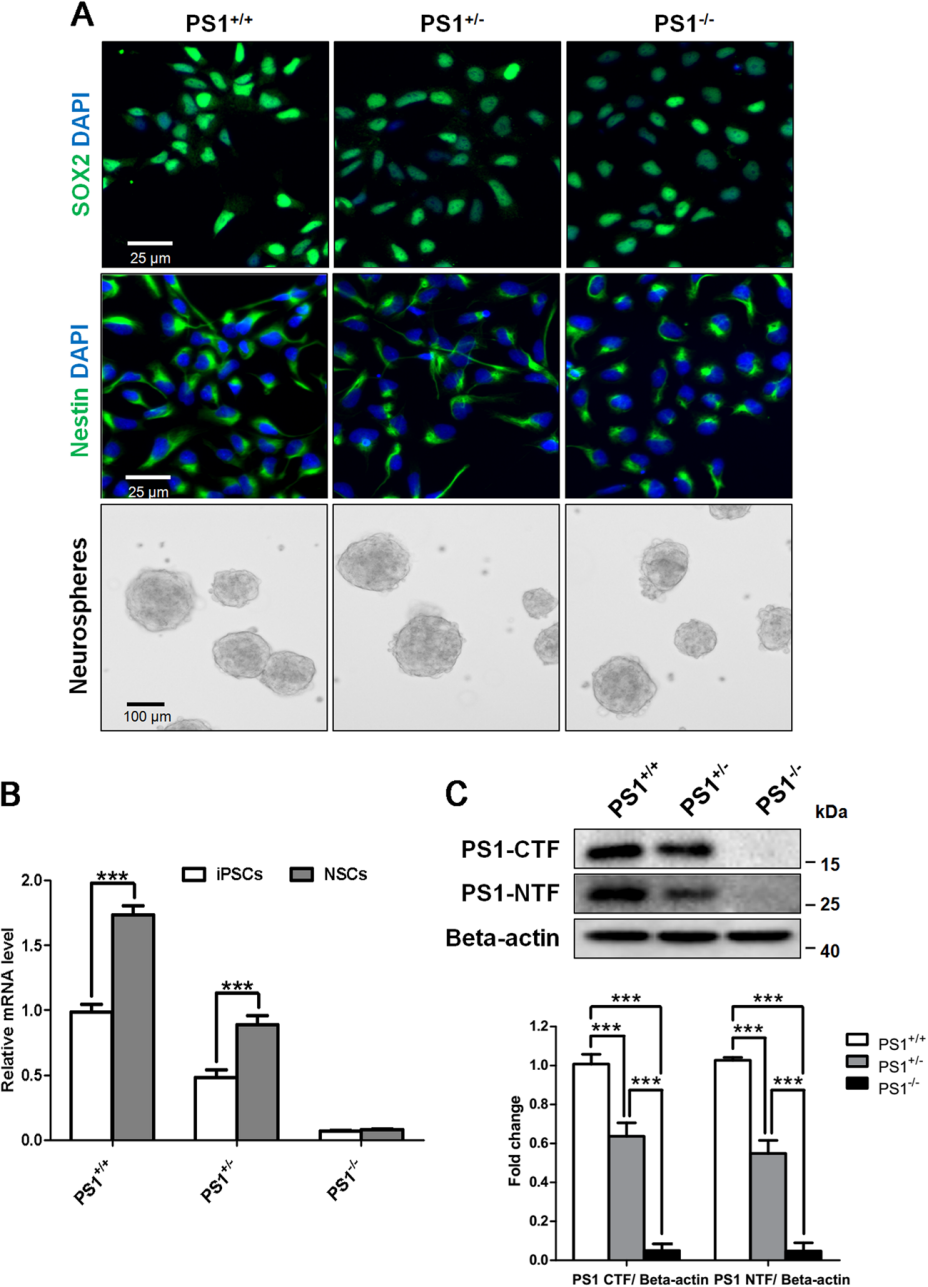
Characteristics of human iPSC-derived NSCs with PS1 knockout. **a** SOX2 and Nestin immunostaining of NSCs from iPSC lines and phase-contrast images of neurospheres. **b** The levels of PS1 mRNA in iPSCs and NSCs were detected by qRT-PCR. **c** The expression of N-terminal fragment (NTF) and C-terminal fragment (CTF) of PS1 was detected and quantified by western blotting using specific antibodies. Data are represented as mean ± SD. ***P < 0.005 was considered significantly different

Using a well-defined protocol for neural differentiation of human iPSCs^30^, NSCs were differentiated from these isogenic iPSCs. All iPSC lines could efficiently generate SOX2-positive and Nestin-positive cells with the same capacity to generate neurospheres (Fig. 1a). The capacity to generate neurospheres is a key criterion for long-term self-renewal of NSCs^31^. The qRT-PCR analysis showed that the PS1 mRNA expression level was upregulated in PS1^+/+^ and PS1^+/^^−^ iPSCs when differentiated into NSCs (Fig. 1b), suggesting that PS1 has a critical role in neural differentiation. The western blot analysis showed that the levels of endoproteolytically cleaved N-terminal and C-terminal fragments of PS1 (NTF and CTF) in PS1-knockout NSCs decreased in a gene copy-dependent manner, compared with the isogenic control (Fig. 1c). Moreover, these isogenic iPSC-derived NSCs were able to efficiently differentiate into neurons (Supplementary Fig. 2).

### PS1 deficiency triggers autophagy suppression in human NSCs

**Fig. 2:**
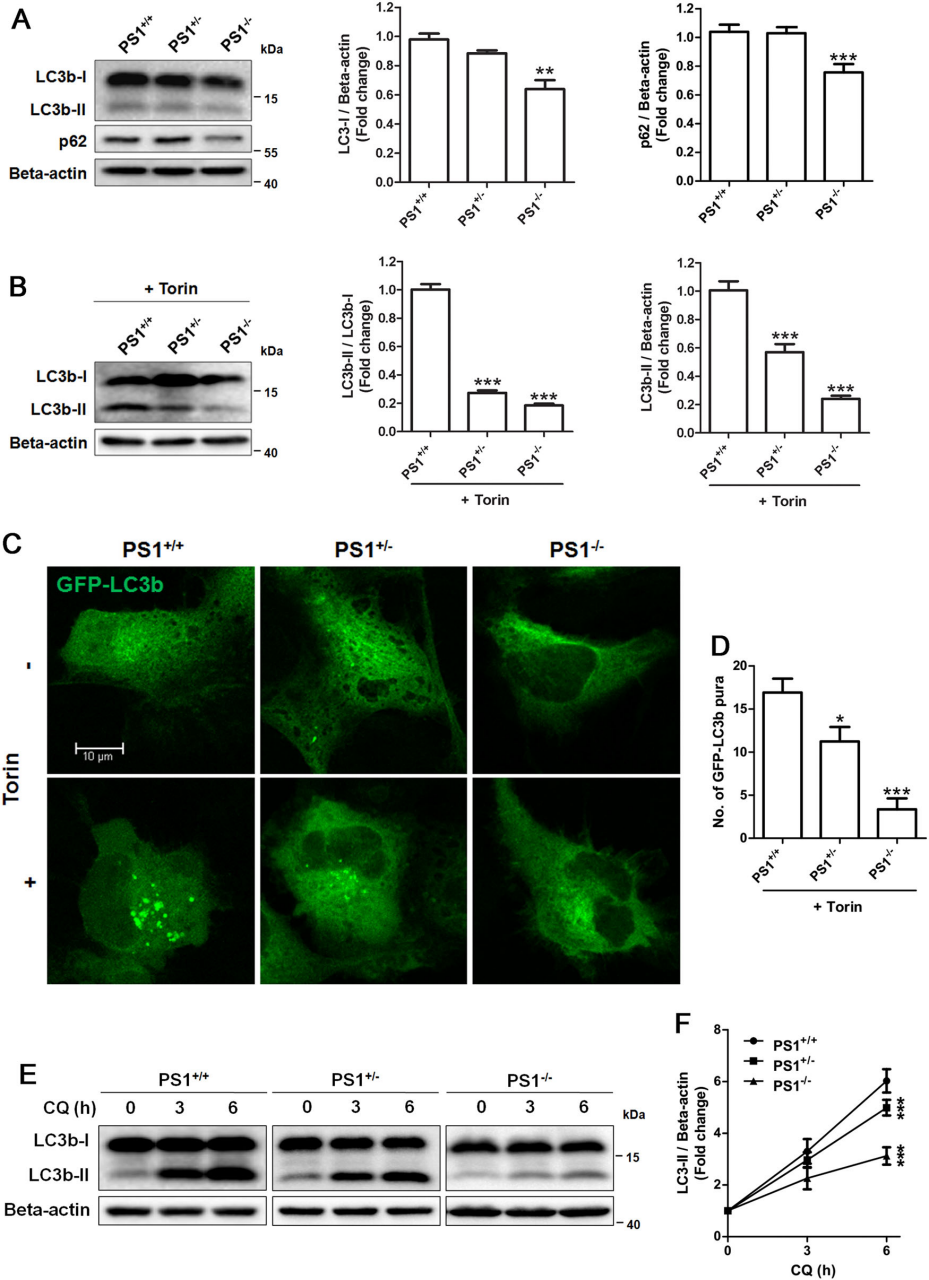
PS1 deficiency suppressed Torin 1-induced autophagosome formation and basal autophagy flux in human NSCs. **a** Western blotting of LC3b and p62 in PS1-knockout NSCs. **b** Western blotting of LC3b and p62 in PS1-knockout NSCs in the presence Torin 1. Autophagy flux and LC3b-II level were quantified by western blotting. **c** Representative images and **d** the number of punctate GFP–LC3 in PS1-knockout NSCs with or without Torin 1 treatment. Sample numbers were *n* = 50. **e** Western blotting of LC3b in PS1-knockout NSCs after incubation with CQ for 0, 3, and 6 h. **f** LC3b-II levels were quantified by western blotting. Beta-actin as internal control. Data are represented as mean ± SD. ***P < 0.005 vs. the PS^+/+^ group was considered significantly different

To determine the autophagy in NSCs, the steady-state level of endogenous LC3b was measured by the western blot analysis. There was a reduction in the expression of LC3b-I and LC3b-II but no increase in autophagy substrate p62 in PS1^−/−^ NSCs, compared with isogenic PS1^+/+^ NSCs (passage 4) (Fig. 2a), showing that autophagy clearance seem to be normal in PS1-knockout NSCs. To investigate the effects of PS1 on mammalian target of rapamycin (mTOR)-mediated autophagy in NSCs, the mTOR inhibitor Torin 1 was used to induce autophagy. Western blotting analysis showed that the ratio of LC3b-II to LC3b-I in PS1^+/+^ NSCs increased by more than fourfold compared with PS1-knockout NSCs in the presence of Torin 1 for 6 h (Fig. 2b). Torin 1 caused a higher level of LC3b-II in PS1^+/+^ NSCs compared with that of either PS1^+/^^−^ or PS1^−/−^ NSCs (Fig. 2b). Furthermore, we used adenovirus expressing green fluorescent protein (GFP)-LC3b (an autophagosome marker) to transfect NSCs, the punctate GFP-LC3b signal substantially increased in PS1^+/+^ NSCs after Torin 1 treatment for 6 h (Fig. 2c). However, Torin 1 treatment did not induce a significant increase in the punctate GFP-LC3b signal in PS1^−/−^ NSCs. As shown in Fig. 2d, the punctate GFP-LC3b signal in PS1^+/−^ and PS1^−/−^ NSCs was significantly less than that in PS^+/+^ NSCs, which was consistent with the western blotting results. The mTOR kinase substrate p70S6K was used to check whether Torin 1 completely inhibited the mTOR activity in PS1-knockout NSCs. Western blot analysis showed that the level of phospho-p70S6K (Thr389) was reduced in all these isogenic NSCs (PS1^+/+^, PS1^+/^^−^, and PS1^−/−^) after Torin 1 treatment for 6 h (Supplementary Figure 3), indicating that the mTOR activity was inhibited as expected in response to Torin 1 in PS1-knockout NSCs.

The autophagy inhibitor chloroquine (CQ) was further used to block the maturation of autophagosomes for determining the basal autophagy flux. As shown in Figs. 2e, f, the level of LC3b-II was dramatically increased in CQ-treated PS1^+/+^ NSCs, whereas the magnitude of the increase of LC3b-II expression was greatly reduced in PS1-deficient cells. These results demonstrated that PS1 deficiency led to the suppression in autophaghic flux of human NSCs, particularly in autophagosome formation.

### PS1 deficiency downregulates the expression of ALP-related genes at transcriptional and translational levels in human NSCs

**Fig. 3:**
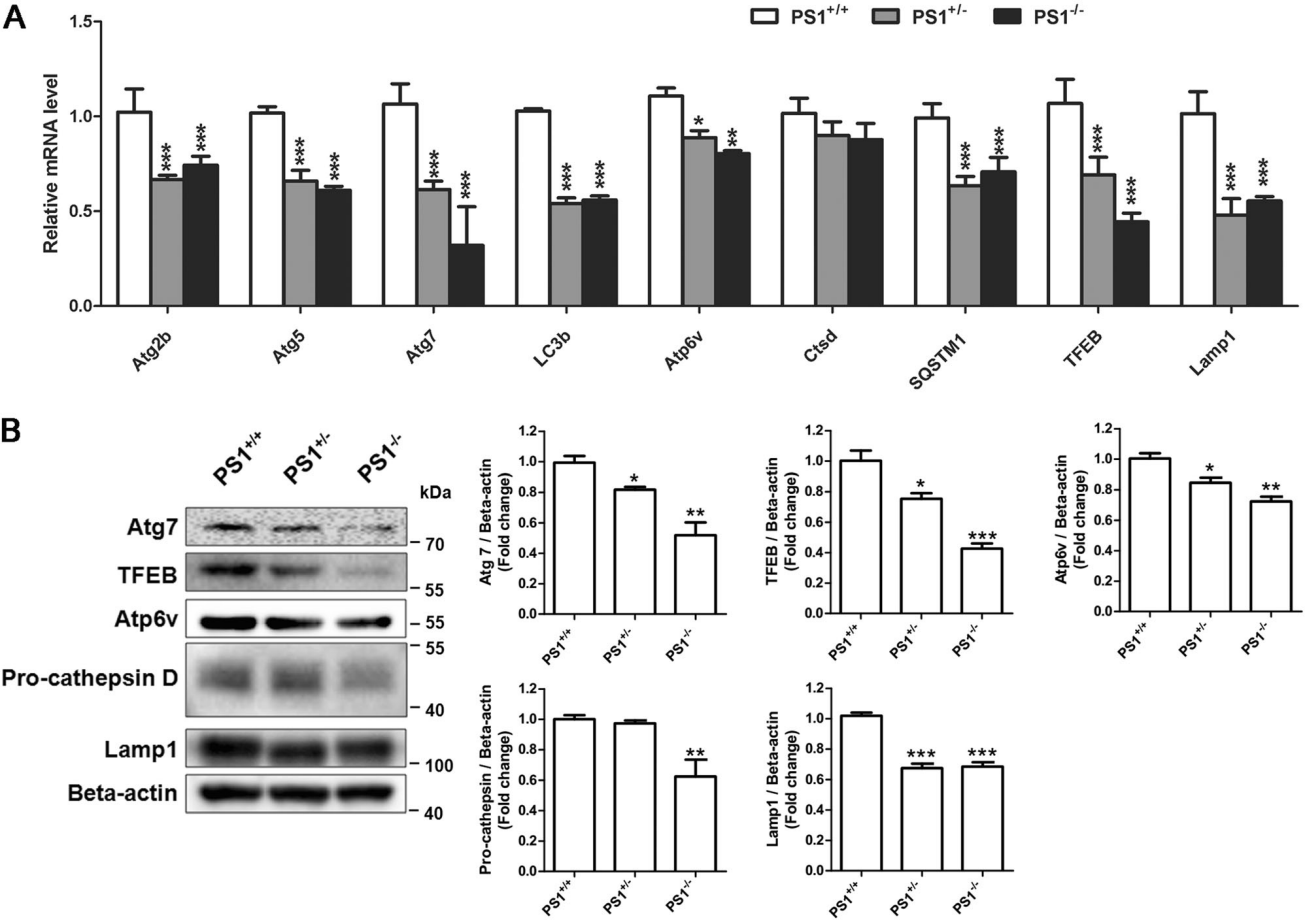
PS1 deficiency downregulated the expression of autophagy-related genes at transcriptional and translational levels in human NSCs. **a** qRT-PCR for determining mRNA expression of autophagy-related genes in PS1-knockout NSCs. **b** The expression of LC3b-1, Atg7, TFEB, Atp6v, pro-cathepsin D, Lamp1, and beta-actin in PS1-knockout NSCs was detected by western blotting with specific antibodies. Data are represented as mean ± SD. *P < 0.05, **P < 0.01, ***P < 0.005 vs. the PS^+/+^ group were considered significantly different

The failure of Torin 1-inducd autophagy initiation and decreased autophagy flux in PS1-deficient NSCs prompted us to measure the basal levels of autophagy-related genes. As shown in Fig. 3a, most of the autophagy-related genes were downregulated in PS1-knockout NSCs. Consistent with the qRT-PCR results, the western blot analysis showed that the expression levels of Atg7, TFEB, Atp6v, pro-cathepsin D, and Lamp1 decreased in PS1-knockout NSCs, suggesting that PS1 is indispensable for maintaining the expression of basal autophagy proteins (Fig. 3b).

### Autophagy suppression in PS1-deficient NSCs is independent of γ-secretase activity

**Fig. 4:**
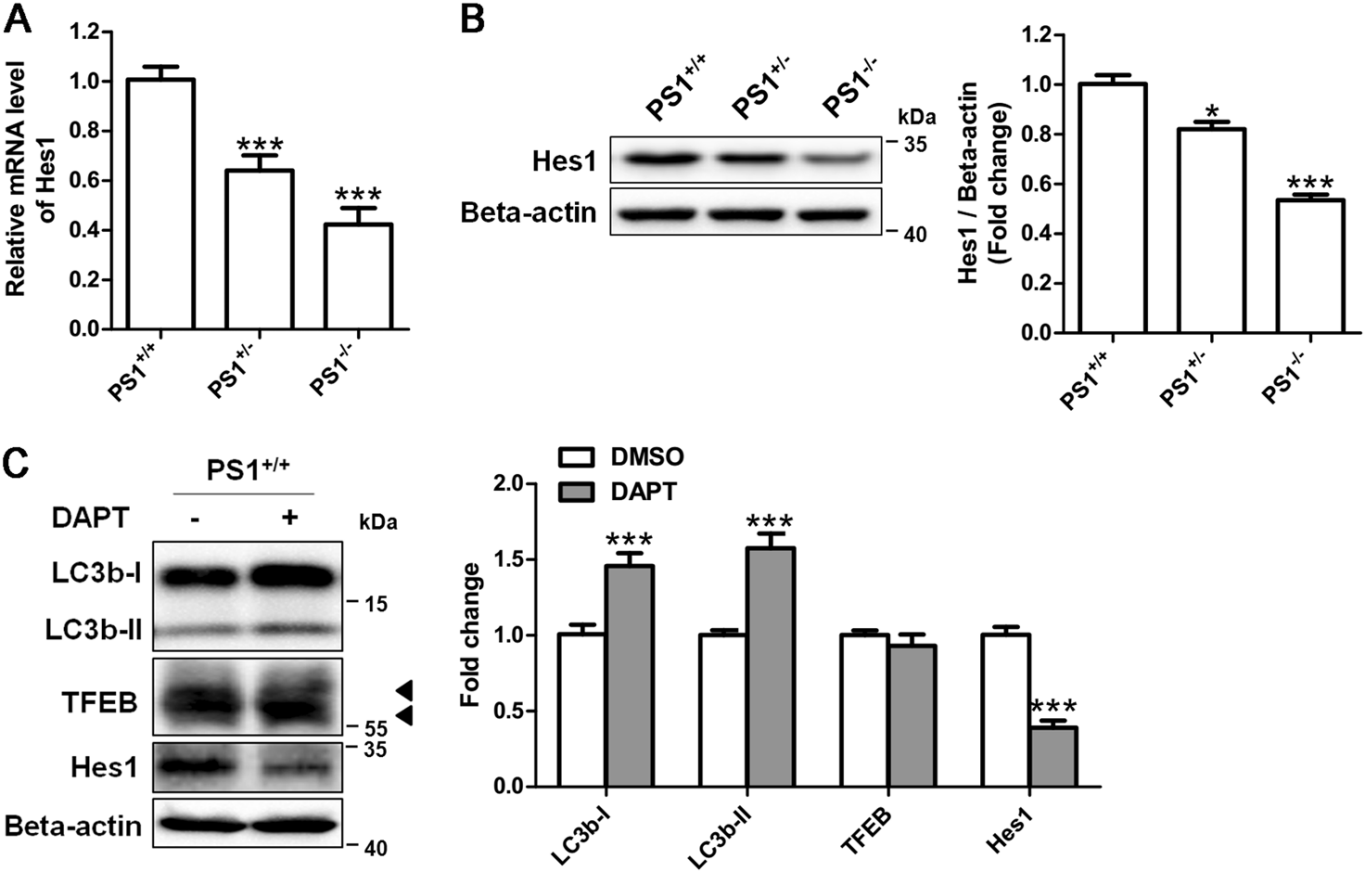
Inhibition of γ-secretase activity did not suppress autophagy in PS1-deficient NSCs. **a** qRT-PCR for determining mRNA expression of Hes1 in PS1-knockout NSCs. **b** Hes1 expression were detected and quantified by western blotting. Data are represented as mean ± SD. *P < 0.05, ***P < 0.005 vs. the PS^+/+^ group were considered significantly different. **c** Western blotting for determining the expression of LC3b and Hes1 in isogenic PS1^+/+^ NSCs were treated with 10 μM DAPT for 24 h. DMSO was used as the vehicle control. ***P < 0.005 vs. the control group were considered significantly different. Data are represented as mean ± SD

The loss of PS1 causes the dysfunction of γ-secretase, because PS1 is a key catalytic component of the γ-secretase complex^32^. Notch signaling is important for maintaining cellular functions of NSCs, whereas inhibition of γ-secretase can reduce this signaling. Thus, we checked the expression of Hes1, a canonical Notch target using qRT-PCR and western blotting. As shown in Figs. 4a, b, the mRNA levels and protein levels of Hes1 were reduced in both PS1^+/−^ and PS1^−/−^ NSCs, suggesting that PS1 deficiency caused dysfunction of γ-secretase and further led to the decrease in Notch signaling in human NSCs. We then assessed whether the γ-secretase inhibitor N-[N-(3,5-Difluorophenacetyl)-L-alanyl]-S-phenylglycine t-butyl ester (DAPT) could mimic the suppressive effects of PS1 deficiency on the expression of autophagy-related proteins. The expression of Hes1 in PS1^+/+^ NSCs was markedly reduced after DAPT treatment for 24 h. However, DAPT treatment elevated LC3b expression levels and generated the band shift of TFEB (Fig. 4c). It was consistent with previous reports that blocking γ-secretase could not impair autophagy function in other models^28,33^. In addition, there was no significant change in LC3b expression in DAPT-treated PS1^−/−^ NSCs compared with vehicle-treated PS1^−/−^ NSCs, suggesting that LC3b expression was not affected by DAPT (Supplementary Figure 4). Thus, these results indicate that autophagy suppression in PS1-deficient NSCs is independent of γ-secretase activity.

### Reduced ERK signaling results in the suppression of autophagy in PS1-deficient NSCs

**Fig. 5:**
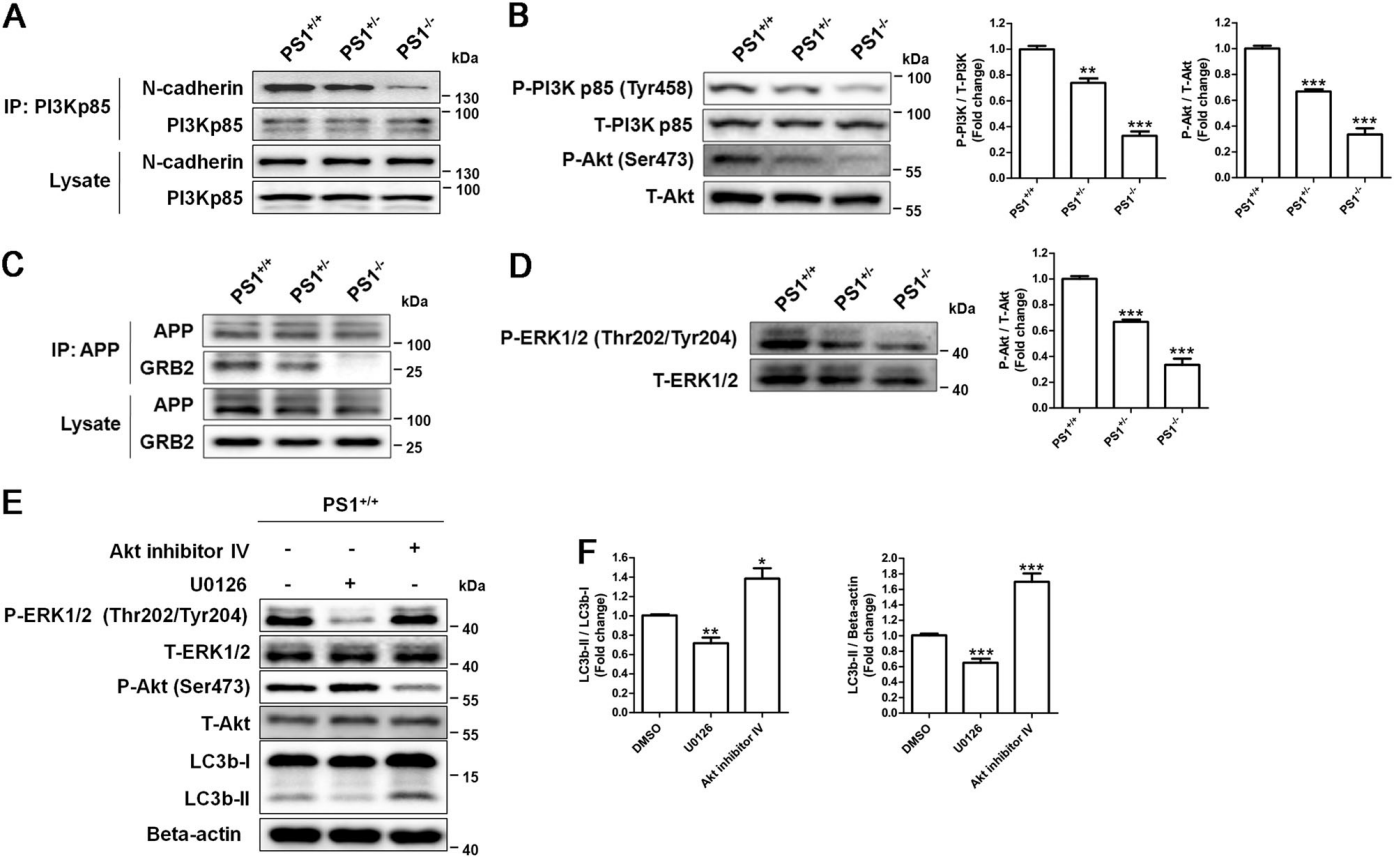
PS1 deficiency-induced autophagy suppression resulted from reduced ERK activity, not reduced Akt activity. **a** The lysates from PS1^+/+^, PS1^+/−^, and PS^−/−^ NSCs were immunoprecipitated with the anti-PI3Kp85 antibody and obtained IPs were analyzed for N-cadherin, PI3Kp85. **b** The expression of P-PI3Kp85, total PI3Kp85, P-Akt, total Akt, and β-actin in PS1-knockout NSCs were determined by western blotting. **c** The lysates from PS1^+/+^, PS1^+/−^, and PS^−/−^ NSCs were immunoprecipitated with the anti-APP antibody and obtained IPs were analyzed for APP, GRB2. **d** The expression of P-ERK1/2, total ERK1/2, and β-actin in PS1-knockout NSCs were determined by western blotting. Data are represented as mean ± SD. **P < 0.01, ***P < 0.005 vs. the PS^+/+^ group were considered significantly different. **e** Western blotting of indicated proteins in PS1^+/+^ NSCs with 10 μM Akt inhibitor IV or 10 μM U0126 treatment. **f** Autophagy flux and LC3b-II level were quantified by western blotting. Data are represented as mean ± SD. ***P < 0.005 vs. the control group was considered significantly different

One of γ-secretase-independent functions of PS1 is to promote the association of N-cadherin with phosphatidylinositol-3-kinase (PI3K), which is a key step for PI3K/Akt activation^10^. Co-immunoprecipitation results revealed that PI3K was associated with both N-cadherin and PS1 in human NSCs (Supplementary Fig. 5). The association of PI3K with N-cadherin was reduced in PS1-deficient NSCs (Fig. 5a). Indeed, western blotting showed that the phosphorylation of PI3K and Akt was dramatically reduced in PS1-knockout NSCs (Fig. 5b). Another γ-secretase-independent function of PS1 is to promote the association of APP with GRB2, which is the upstream of the ERK signaling^34^. Co-immunoprecipitation results revealed that APP interacted with GRB2 in human NSCs (Supplementary Figure 5), but the association of APP with GRB2 was impaired in PS1-deficient NSCs (Fig. 5c). We found that the phosphorylation of ERK was also dramatically reduced in PS1-knockout NSCs (Fig. 5d). We then used the Akt inhibitor (Akt inhibitor IV) and the ERK inhibitor (U0126) to test whether blocking two signaling pathways suppressed autophagy in NSCs. Western blotting analysis showed that PS1^+/+^ NSCs treated with U0126 reduced the conversion of LC3b and the level of LC3b-II, exhibiting a same expression profile of autophagy-related proteins as PS1-deficient NSCs (Fig. 5d–f). In contrast, Akt inhibitor IV enhanced the conversion of LC3b and the level of LC3b-II in PS1^+/+^ NSCs (Fig. 5d–f), suggesting that the ERK signaling might account for the decrease in autophagosome formation in PS1-deficient NSCs.

### Glycogen synthase kinase 3 beta (GSK3β) inhibitor promotes autophagy in PS1-deficient NSCs via enhancing CREB/TFEB signaling

**Fig. 6:**
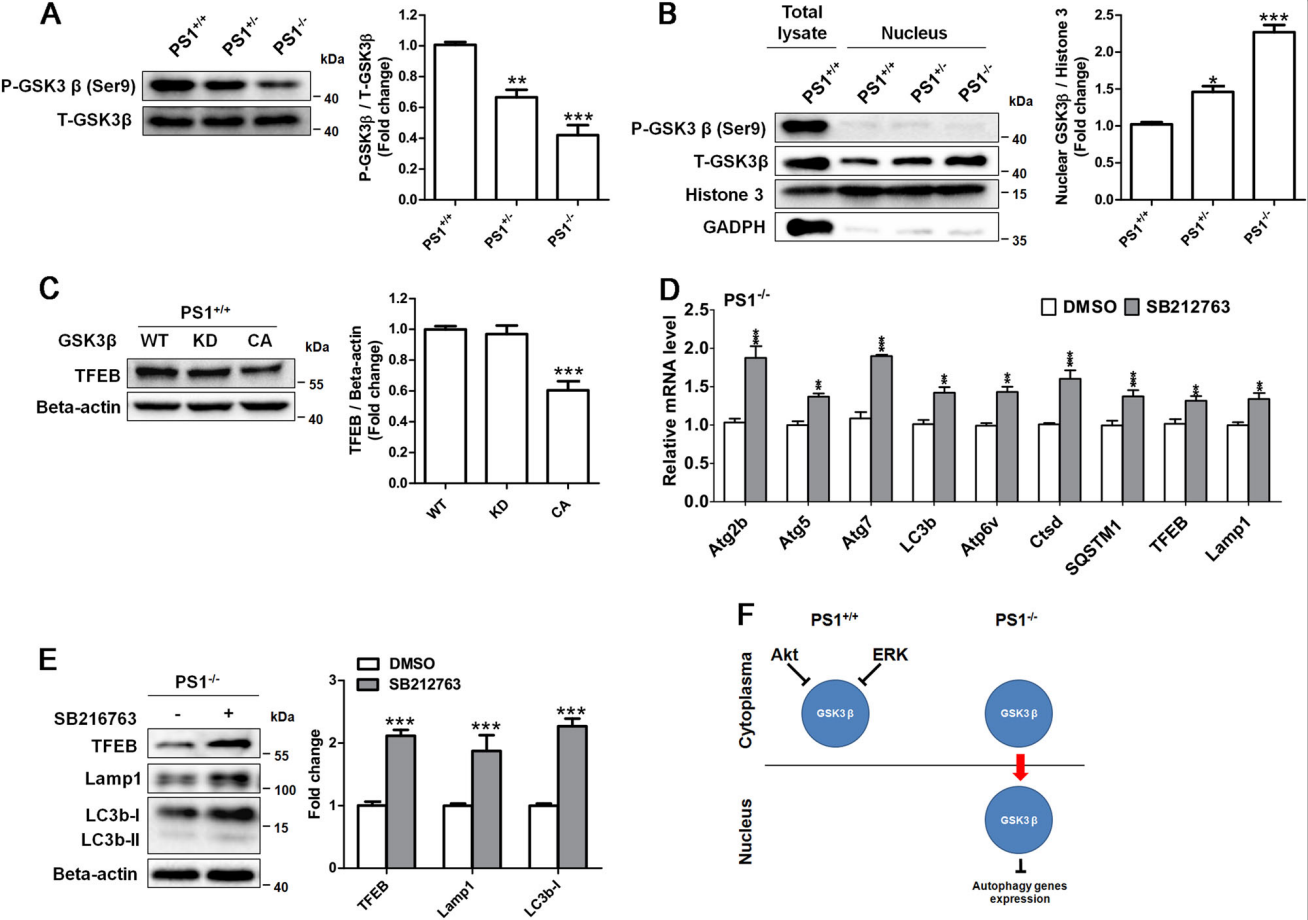
GSK3β inhibition upregulated the expression of autophagy proteins in PS1-knockout NSCs. **a** The expression of P-GSK3β and total GSK3β in PS1-knockout NSCs were determined by western blotting. **b** The levels of nuclear GSK3β in PS1-knockout NSCs were evaluated by western blotting. Data are represented as mean ± SD. *P < 0.05, **P < 0.01, ***P < 0.005 vs. the PS1^+/+^ group was considered significantly different. **c** The expression of TFEB in PS1^+/+^ NSCs expressing GSK3β (WT), GSK3β (KD), or GSK3β (CA) was determined by western blotting. ***P < 0.005 vs. the GSK3β (WT) group was considered significantly different. **d** qRT-PCR for determining mRNA expression of autophagy-related genes in PS1^−/−^ NSCs treated with 10 μM SB212763 for 4 h. DMSO was used as the vehicle control. **e** After NSCs were treated with 10 μM SB212763 for 24 h, the expression of TFEB, Lamp1, LC3b-I, and β-actin in PS1^−/−^ NSCs was determined by western blotting with specific antibodies. Data are represented as mean ± SD. *P < 0.05, **P < 0.01, ***P < 0.005 vs. the control group was considered significantly different. **f** A proposed mechanism of PS1 deficiency-induced GSK3β nuclear translocation to block the expression of autophagy genes

PS1 has been described to have an indirect role for regulating GSK3β activity^10^. In addition, GSK3β is one of downstream targets of Akt and ERK1/2 signaling. Thus, we tested the involvement of GSK3β in autophagy defects of PS1-deficient NSCs. Western blotting analysis showed that the levels of phospho-GSK3β (Ser9) was decreased in PS1-knockout NSCs, suggesting that the PS1 deficiency caused the activation of GSK3β (Fig. 6a). Moreover, increased expression of GSK3β was observed in the nuclear fraction of PS1-knockout NSCs (Fig. 6b), showing that activated GSK3β was translocated to the nucleus in NSCs. To determine whether TFEB expression was affected by GSK3β in NSCs, we transiently transfected PS1^+/+^ NSCs with a plasmid expressing the GSK3β mutant. We found that overexpression of constitutively active GSK3β (CA) reduced the level of TFEB, whereas overexpression of GSK3β kinase-dead had no effect on the TFEB expression level, compared with wild-type GSK3β (Fig. 6c), suggesting an inhibitory effect of GSK3β activation on TFEB expression in PS1-deficient NSCs. GSK3β is considered as a therapeutic target for AD. Thus, we tested the effect of GSK3β inhibition on the expression levels of autophagy-related mRNA and proteins in PS1^−/−^ NSCs. The qRT-PCR results showed that the basal levels of autophagy-related genes were enhanced by the treatment of the specific GSK3β inhibitor SB216763 in PS1^−/−^ NSCs (Fig. 6d). Moreover, the western blotting analysis demonstrated that treatment with SB216763 for 24 h enhanced the protein levels of TFEB, Lamp1, and LC3b (Fig. 6e), suggesting that nuclear GSK3β could block the expression of autophagy-related genes in PS1-deficient NSCs (Fig. 6f).

**Fig. 7:**
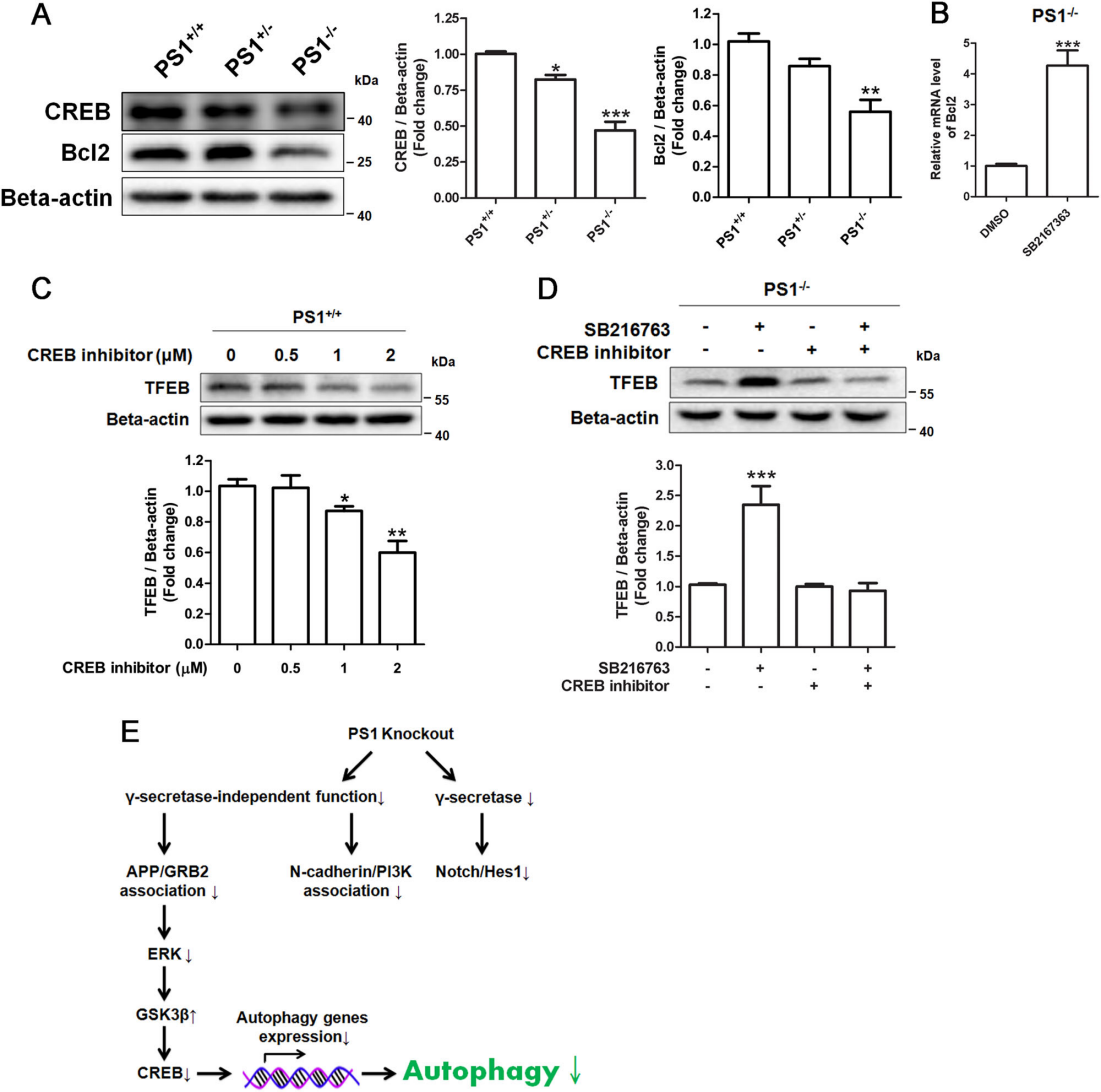
CREB inhibitor blocked the effect of GSK3β in PS1-knockout NSCs. **a** The expression of CREB and Bcl2 in PS1-knockout NSCs were determined by western blotting. Data are represented as mean ± SD. *P < 0.05, **P < 0.01, ***P < 0.005 vs. the PS1^+/+^ group was considered significantly different. **b** qRT-PCR for determining mRNA expression of Bcl2 in PS1^−/−^ NSCs treated with 10 μM SB212763 for 4 h. DMSO was used as the vehicle control. Data are represented as mean ± SD. ***P < 0.005 vs. the control group was considered significantly different. **c** The effect of CREB inhibitor on TFEB expression in PS^+/+^ NSCs was determined by western blotting. Data are represented as mean ± SD. **P < 0.01, ***P < 0.005 vs. the control group was considered significantly different. **d** PS^−/−^ NSCs were treated with 10 μM SB216763 or 2 μM CREB inhibitor or both for 24 h. The expression of TFEB and beta-action were determined by western blotting. Data are represented as mean ± SD. ***P < 0.005 vs. control group was considered significantly different. **e** A proposed mechanism of PS1 deficiency-induced autophagy suppression through the reduced ERK signaling

Active GSK3β could block CREB, which is a critical transcription factors for positively regulating the expression of autophagy genes^23,35^. We found that levels of CREB and its downstream gene Bcl2 are decreased in PS1-knockout NSCs, suggesting that PS1 deficiency caused the decrease of CREB activity (Fig. 7a). The CREB inhibitor 666–15 is recently identified to block the interaction between the KID domain of CREB and the KIX domain of CBP^36^. We found that the CREB inhibitor effectively reduced the TFEB level in PS1^+/+^ NSCs in a concentration-dependent manner (Fig. 7b), suggesting that CREB positively regulate the expression of TFEB in NSCs. In PS1^−/−^ NSCs, GSK3β inhibition enhanced the levels of TFEB, whereas this effect was reduced by the CREB inhibitor (Fig. 7c), suggesting that the CREB/TFEB pathway contributed to the positive effect of GSK3β inhibitor on autophagy in PS1-deficient NSCs.

## Discussion

PS1 has been reported as a neuroprotective protein^10–12^ and its expression in the brain gradually decreases with age^5,6^, suggesting that the PS1 level is a key factor in the development of AD. In the present study, we created human iPSCs with heterozygous and homozygous PS1 deletion via CRISPR/Cas9-based gene editing and demonstrated that the normal level of PS1 is essential for maintaining autophagy in human iPSC-derived NSCs. PS1 deficiency reduced expression levels of autophagy-related proteins in NSCs, which led to the suppression of autophagy initiation and autophagy flux. Our study provides evidence that autophagy suppression in NSCs, which results from the loss of PS1 function may be one of causes for early-onset AD pathogenesis.

Serious autophagy defects such as accumulation of autophagic vacuoles and lysosomal dysfunction occur in PS1-deficient neurons, but not in PS1-deficient ESCs. In PS1-deficient NSCs, we found reduced expression levels of autophagy-related proteins, which led to the suppression of autophagy initiation and autophagy flux. Some functions of NSCs such as self-renew and neurogenesis need basal autophagy^37,38^. In addition, a previous study reported that neurogenesis was reduced in forebrain-specific PS1-knockout mice^39^. Once autophagy is suppressed, it may gradually lead to various sequential damages such as the increase of ROS and mitochondria dysfunction, and thereby may cause abnormal neurogenesis and loss of stemness^40,41^. In addition, NSCs are found in the adult hippocampus where impairments often take place in the early stages of human AD^42^. Thus, the results of our study help understand the mechanisms underlying autophagy defects in AD.

An interesting finding of our study is that PS1-deficient NSCs were able to differentiate into neurons without dominant impairments, which is inconsistent with impaired neurogenesis found in PS1-knockout mice^42^. The exact mechanisms underlying the difference in neurogenesis between PS1-knockout mice and cultured PS1-deficient NSCs remain unknown. We proposed that neuronal differentiation of iPSC-derived NSCs is an in vitro model and their differentiation is undertaken under a well-controlled condition supplied with sufficient nutrients such as GDNF, BDNF, NT3, ascorbic acid, and cAMP, which may compromise the impairments of PS1 deficiency on neurogenesis. However, future studies on exploring the exact mechanisms underlying the difference in neurogenesis between PS1-deficient in vivo models and in vitro models are absolutely necessary.

PS1 exhibits γ-secretase-dependent and -independent functions, which involve in various biological events through multiple pathways. In this study, a Notch downstream target Hes1 decreased in PS1-deficient NSCs, which was consistent with a previous report that PS1 deficiency caused the dysfunction of γ-secretase and thereby blocks Notch signaling^43^. However, γ-secretase inhibitor DAPT could downregulate the level of Hes1 but did not decrease LC3b in PS1^+/+^ NSCs, revealing that the loss of γ-secretase function of PS1 did not account for autophagy defect in PS1-deficient NSCs. On the other hand, previous studies have stated that the roles of PS1 in regulating PI3K/Akt and ERK signaling are γ-secretase-independent^10,34^. Our study found that both PI3K/Akt and ERK signaling were inhibited in PS1-deficient NSCs. Only inhibited ERK signaling causes the reduction in LC3b. The ERK signaling has been reported to positively regulate autophagy, whereas blocking ERK signaling reduced TFEB activity^44^, which is consistent with our results that the loss of ERK signaling induces autophagy defects in PS1-deficient NSCs. In addition, we found APP could interact with the adaptor GRB2, whereas their association was decreased in PS1-knockout NSCs. This APP/GRB2 complex is important to modulate the activity of ERK^34^. In addition to γ-secretase, only GRB2/ERK signaling is the crosstalk between APP and PS1, which may implicate in AD development.

Recent studies indicate that inhibiting GSK3β enhances lysosomal biogenesis and autophagic degradation via increasing the nuclear translocation of TFEB, whereas overexpression of CA GSK3β reduced the nuclear TFEB and its expression^45–47^. It should be noted that overexpression of active GSK3β cannot represent the cellular physiological role of active GSK3β if the effects of upstream kinases such as Akt or ERK are ignored. In addition, active GSK3β could block CREB, which is a critical transcription factor for upregulating the expression of bcl2, ALP genes, and TFEB^23,35^. In this study, both transcription factors CREB and TFEB simultaneously were downregulated by active GSK3β in PS1-knockout NSCs. In addition, GSK3β increased in the nucleus of PS1-knockout NSCs, implying that nuclear GSK3β may reduce CREB transcriptional activity. Our result further demonstrated that pharmacological inhibition of GSK3β resulted in TFEB increase, whereas this effect could be blocked by CREB inhibitor, revealing active GSK3β led autophagy suppression in PS1-deficient NSCs via reducing CREB/TFEB pathway.

In summary (Fig. 7d), PS1 deficiency inhibits a number of signaling pathways such as Notch/Hes1, N-cadherin/PI3K/Akt, and APP/GRB2/ERK signaling. ERK signaling inhibition was found to cause autophagy suppression. Our results support that GSK3β could be a potential target for improving PS1 deficiency-induced autophagy defects through activating ERK/CREB. The results obtained from these PS1-deficient models could translate to the more common forms of AD and help understand the mechanisms underlying autophagy defects in AD.

## Materials and Methods

### Materials

For western blotting, PS1-CTF (Cat#3622), actin (Cat#4967), GADPH (Cat#5174), LC3B (Cat#12741), Bcl2 (Cat#1507), Atg7 (Cat#8558), TFEB (Cat#4240), LAMP1 (Cat#9091), P-p70S6K (Cat#9205), Histone H3 (Cat#9715), P-Akt (Cat#4060), Akt (Cat#9272), P-ERK (Cat#9101), ERK (Cat#9102), P-GSK3β (Cat#9336), GSK3β (Cat#12456), Grb2 (Cat#3972), N-cadherin (Cat#14215), P-PI3Kp85 (Cat#4228), and PI3Kp85 (Cat#4257) antibodies were purchased from Cell Signaling Technology. PS1-NTF antibody (Cat#MAB1563) was from Millipore. APP (Cat# SIG-39188) was from BioLegend. For immunostaining, Nestin (Cat#ab22035) and TFEB antibody (Cat#ab122910) were from Abcam. OCT4 (Cat#sc-5279) was from Santa Cruz. Sox2 (Cat#2460) was from Stemgent. TUJ1 (Cat#T8578) was from Sigma. In addition, Torin 1, Y27632, Akt inhibitor IV, U0126, and SB216763 were obtained from Calbiochem (San Diego, CA, USA). CQ was purchased from Sigma.

### Human UC-iPSC generation and characterization

The urine cells were reprogrammed as described previously^29^. In brief, urine cells were collected from a healthy donor with informed consent. The purposes and processes for collecting urine cells and generating iPSCs were explained to the donor in great detail. A total of ~500 ml of a mid-stream urine sample was collected from the donor and centrifuged to collect the exfoliated cells. The primary urine cells were cultured in urine cell medium consisting of Dulbecco’s modified Eagle’s medium/F12 medium (Gibco, Grand Island, NY, USA) supplemented with 10% fetal bovine serum, 1 mM GlutaMAX (Life Technologies, Carlsbad, CA, USA), 0.1 mM non-essential amino acids, 0.1 mM β-mercaptoethanol, and a SingleQuot Kit (CC-4127 REGM; Lonza, Portsmouth, NH, USA). After amplifying the urine cells, a pCEP4 episomal vector containing the SOX2, OCT4, KLF4, and SV40LT genes^48^, and the other pCEP4 vector carrying a miR302–367 precursor^49^ were co-transfected into urine cells by nucleofection (Amaxa Basic Nucleofector Kit for primary mammalian epithelial cells, T-013 program; Lonza). The transfected urine cells were plated on Matrigel (BD Biosciences, San Diego, CA, USA)-coated six-well plates (1–3 × 10^5^ cells per well) in urine cell culture medium for the first 2 days, then the media was changed to mTeSR1 (Stemcell Technologies, Cambridge, MA, USA). The medium was exchanged every 2 days until 15 days after transfection, then colonies were picked up and transferred onto a new Matrigel plate with mTeSR1. For further cell expansion, the cells were dissociated into small clusters or single cells. Expression of the pluripotency genes Nanog and Oct4 was analyzed by real-time PCR, compared with the H9 human ESC line. Expression of the pluripotent markers Nanog and Oct4 was confirmed by immunofluorescence. Karyotype analysis was performed to identify whether UC-iPSCs had a normal karyotype.

### Generation of PS1-knockout iPSC lines

gRNA was designed as 5′-GN20NGG-3′, according to a protocol described previously to generate PS1-knockout cells^50^. UC-iPSCs were transfected with a mixture of Cas9 and gRNA with Lipofectamine 3000. Approximately 24 h after recovery, the cells were selected using 50 μg/mL G418 for positive selection. Next, the cells were re-plated as single cells into 96-well plates in the presence of Y27632. The cell colonies were subcultured and part of each colony was collected for qRT-PCR to check PS1 levels.

### NSCs induction and culture

NSCs were generated from iPSCs as described previously with some modifications^30^. In brief, iPSCs were split as cell clumps and plated on 6-well tissue culture plates coated with Matrigel in PSC Neural Induction Medium (Life Technologies) with Y27632, at a density of 2.5–3.0 × 10^4^ cells/cm^2^. The Neural Induction Medium was changed every other day from days 1 to 5. After day 5, the Neural Induction Medium was changed every day until day 7. The cells were dissociated on day 7 using Accutase, re-suspended in Neural Induction Medium containing 5 μM Y27632, and plated on Matrigel-coated dishes at 1.2 × 10^5^ cells/cm^2^. The Neural Induction Medium was changed every other day until the NSCs were 90% confluent. The NSCs were cryopreserved in Neural Induction Medium containing 10% dimethyl sulfoxide (DMSO) (Sigma-Aldrich, St. Louis, MO, USA). Passage 4 NSCs were used in all the experiments to ensure the stability.

### Neurosphere formation

The attached NSCs were dissociated with Accutase to collect the neurospheres. Single NSCs were seeded at a density of 5 × 10^4^ in an untreated 12-well plate with Neural Induction Medium and incubated at 37 °C in 95% air and 5% CO_2_. The medium was replaced every other day.

### qRT-PCR analysis

Total RNA was isolated using RNA-Solv Reagent (OMEGA). Reverse transcription was performed with 2 μg RNA using ReverTra Ace (TOYOBO) and Oligo (dT) 18 (TaKaRa). qRT-PCR was carried on SYBR® Premix Ex Taq (TaKaRa) using ViiA™ 7 Real-Time PCR System (Thermo). Reaction procedures were listed as follow: an initial step at 95 °C for 5 min, 40 cycles of 94 °C for 15 s, 60 °C for 34 s. The primers were shown in Supplementary Table 1.

### GFP-LC3b quantification

Adenovirus expressing GFP-LC3b (Beyotime, Shanghai, China) was transiently transfected into NSCs. The cells were treated with Torin 1 (1 μM in DMSO; Calbiochem), or DMSO (vehicle control). After treatment for 6 h, the cells were fixed in 4% paraformaldehyde at room temperature for 20 min. After washing with Dulbecco's phosphate-buffered saline (DPBS), fluorescence was visualized and photographed using a Leica confocal microscope. Quantification of EGFP-LC3 was performed using ImageJ 1.50b bundled with Java 1.8.0_60 software (National Institutes of Health, MA, USA) for measuring the number and size of EGFP-LC3 puncta. Cells expressing high levels of EGFP-LC3 were excluded to avoid counting GFP aggregates.

### Immunostaining

The cells for immunostaining were fixed in 4% paraformaldehyde at room temperature for 20 min, blocked with 5% donkey serum in 0.3% Triton™ X-100 for 1 h, and then incubated with primary antibodies overnight at 4 °C. After washing three times with DPBS, the cells were incubated at room temperature for 1 h with fluorophore-conjugated secondary antibodies (Life Technologies) against the immunoglobulin of the species from which the primary antibody was generated. Upon completion of immunostaining, the cells were briefly stained with 4′,6-diamidino-2-phenylindole to reveal the cell nuclei. After washing with DPBS, fluorescence was visualized and photographed using an In Cell Analyzer 2000 (GE Healthcare, Parsippany, NJ, USA) or by Leica confocal microscopy.

### Immunoprecipitation

Immunoprecipitation of β-cadherin was performed as described previously^10^. In brief, cells were lysed in TNE buffer (25 mM Tris–HCl [pH 7.6], 150 mM NaCl, 1 mM EDTA) containing 1% Triton X-100 plus protease inhibitor and phosphatase inhibitor cocktails (Thermo Fisher Scientific, Waltham, MA, USA). Following centrifugation at 17,000 × *g*, supernatants (4 mg/ml protein) were treated with Protein A/G magnetic beads (Thermo Fisher Scientific) for 2 h. Beads were removed and supernatants were incubated with primary antibody overnight at 4 °C, and then treated with magnetic beads for 2 h. After washing with Tris-buffered saline plus 0.2% NP-40, Proteins in beads were eluted in 1 × sample buffer and analyzed using western blotting.

### Western blotting analysis

Cells in culture plates were rinsed once with ice-cold DPBS and lysed in RIPA buffer containing 1% phenylmethylsulfonyl fluoride and 1% protease/phosphatase inhibitor cocktail (Thermo Fisher Scientific) for 30 min at 4 °C, followed by centrifugation at 12,500 × *g* for 20 min at 4 °C. Lysates in 1 × sample buffer were boiled for 5 min at 95 °C for denaturation and separated by SDS-polyacrylamide gel electrophoresis. The target proteins were detected by western blotting with their respective specific antibodies, and β-actin was used as an internal control. The blot was visualized using an ECL kit (GE Healthcare) according to the manufacturer’s instructions. The intensity of the bands was quantified using Image Lab 5.0 software.

### Torin 1, CQ, and GSK3β inhibitor treatments

To induce autophagy, NSCs were treated with 1 μM Torin 1 for 6 h. To block autophagy, NSCs were treated with 30 μM CQ for 6 h to rescue the expression of autophagy-related mRNAs and proteins, NSCs were treated with 10 μM of the GSK3β inhibitor SB216763 for 4 and 24 h respectively.

### Statistical analysis

The statistical analysis was performed using GraphPad Prism 5.0 statistical software (GraphPad Software, Inc., San Diego, CA, USA). All experiments were performed in triplicate. Data were expressed as mean ± SD. Statistical analysis was carried out using one-way analysis of variance followed by Tukey’s multiple comparison or two-sided Mann–Whitney *U*-test for two group. A *p*-value < 0.05 was considered significant.

## Electronic supplementary material

MATERIAL Supplementary figure legends and table

Supplementary Figure 1

Supplementary Figure 2

Supplementary Figure 3

Supplementary Figure 4

Supplementary Figure 5
