# Supplementary material for: Presenilin 1 deficiency suppresses autophagy in human neural stem cells through reducing γ-secretase-independent ERK/CREB signaling
Source: Cell Death Dis. 2018 Aug 29;9(9):879. doi: 10.1038/s41419-018-0945-7 (PMC6115391; doi:10.1038/s41419-018-0945-7)
Supplement: Supplementary file 1 — MATERIAL Supplementary figure legends and table [file 41419_2018_945_MOESM1_ESM.docx]

**Supplementary Figure Legends:**

**Supplementary Figure 1.** (A) Schematic diagram showing sgRNA target site for generation of human PS1 knockout iPSCs by CRISPR/Cas 9 system. (B) Sanger sequencing of human PS1 knockout iPSCs. (C) The levels of PS1 mRNA in human PS1 knockout iPSCs were detected by qRT-PCR. Data are represented as mean ± SD. ***P < 0.005 vs. PS1^+/+^ group was considered significantly different. (D) OCT4 immunostaining of human PS1 knockout iPSCs. Scale bar: 100 μm. (E) Normal male karyotype of human PS1 knockout iPSCs.

**Supplementary Figure 2.** The phenotypes in PS1 knockout iPSCs-derived neurons. (A) Immunofluorescence staining of specific markers TUJ-1 for neurons. (B) Mutation dependent changes in total APP in PS1 knockout iPSCs-derived neurons. Data are represented as mean ± SD. ***P < 0.005 vs. PS1^+/+^ group was considered significantly different. Scale bar: 75 μm.

**Supplementary Figure 3.** After NSCs were treated with or without Torin 1, P-p70S6K and actin expression were measured and quantified by western blotting. Data are represented as mean ± SD. ***P < 0.005 vs. no treatment group was considered significantly different.

**Supplementary Figure 4.** Western blotting for determining the expression of LC3b in isogenic PS1^-/-^ NSCs with 10 μM DAPT treatment for 24 h. DMSO was used as the vehicle control.

**Supplementary Figure 5.** The interaction between N-cadherin and PI3Kp85, or APP and GRB2 in NSCs. (A) Total lysates from PS1^+/+^ NSCs were immunoprecipitated with anti-PI3Kp85 antibody. N-cadherin, PI3Kp85, and PS1-CTF were analyzed by western blotting. (B) Total lysates from PS1^+/+^ NSCs were immunoprecipitated with anti-APP antibody. APP and GRB2 were analyzed by western blotting.

**Supplementary tables:**

**Supplementary table 1: Real-time PCR Primers for markers of iPSC**

| **Primer** | **Sequence (5’-3’)** |
| --- | --- |
| GAPDH-qPCR-F | AGCTGAACGGGAAGCTCACT |
| GAPDH-qPCR-R | AGGTCCACCACTGACACGTTG |
| PS1-qPCR-F1 | TGACTCTCTGCATGGTGGTGG |
| PS1-qPCR-R1 | TCTCTGGCCCACAGTCTCGGT |
| Atg2b-qPCR-F1 | GGCAGTAGCTTTCTTTACTTGTATA |
| Atg2b-qPCR-R1 | GTACCTATAAATCTAAGGTGATCGT |
| Atg5-qPCR-F1 | GCAAGCCAGACAGGAAAAAG |
| Atg5-qPCR-R1 | GACCTTCAGTGGTCCGGTAA |
| Atg7-qPCR-F1 | ACCCAG AAGAAGCTGAACGA |
| Atg7-qPCR-R1 | CTCATTTGCTGC TTGTTCCA |
| LC3B-qPCR-F1 | ACCATGCCGTCGGAGAAG |
| LC3B-qPCR-R1 | ATCGTTCTATTATCACCGGGATTTT |
| ATP6V-qPCR-F1 | GAGGGGCAGATCTATGTGGA |
| ATP6V-qPCR-R1 | GCATGATCCTTCCTGGTCAT |
| Ctsd-qPCR-F1 | TTGCTGTTTTGTTCTGTGGTTTTC |
| Ctsd -qPCR-R1 | CAGACAGGCAGGCAGCATT |
| SQSTM1-qPCR-F1 | CTGGGACTGAGAAGGCTCAC |
| SQSTM1-qPCR-R1 | GCAGCTGATGGTTTGGAAAT |
| TFEB-qPCR-F1 | GTCCGAGACCTATGGGAACA |
| TFEB-qPCR-R1 | CGTCCAGACGCATAATGTTG |
| Lamp1-qPCR-F1 | ACGTTACAGCGTCCAGCTCAT |
| Lamp1-qPCR-R1 | TCTTTGGAGCTCGCATTGG |
