# Supplementary figures and images for: Presenilin 1 deficiency suppresses autophagy in human neural stem cells through reducing γ-secretase-independent ERK/CREB signaling
Source: Cell Death Dis. 2018 Aug 29;9(9):879. doi: 10.1038/s41419-018-0945-7 (PMC6115391; doi:10.1038/s41419-018-0945-7)

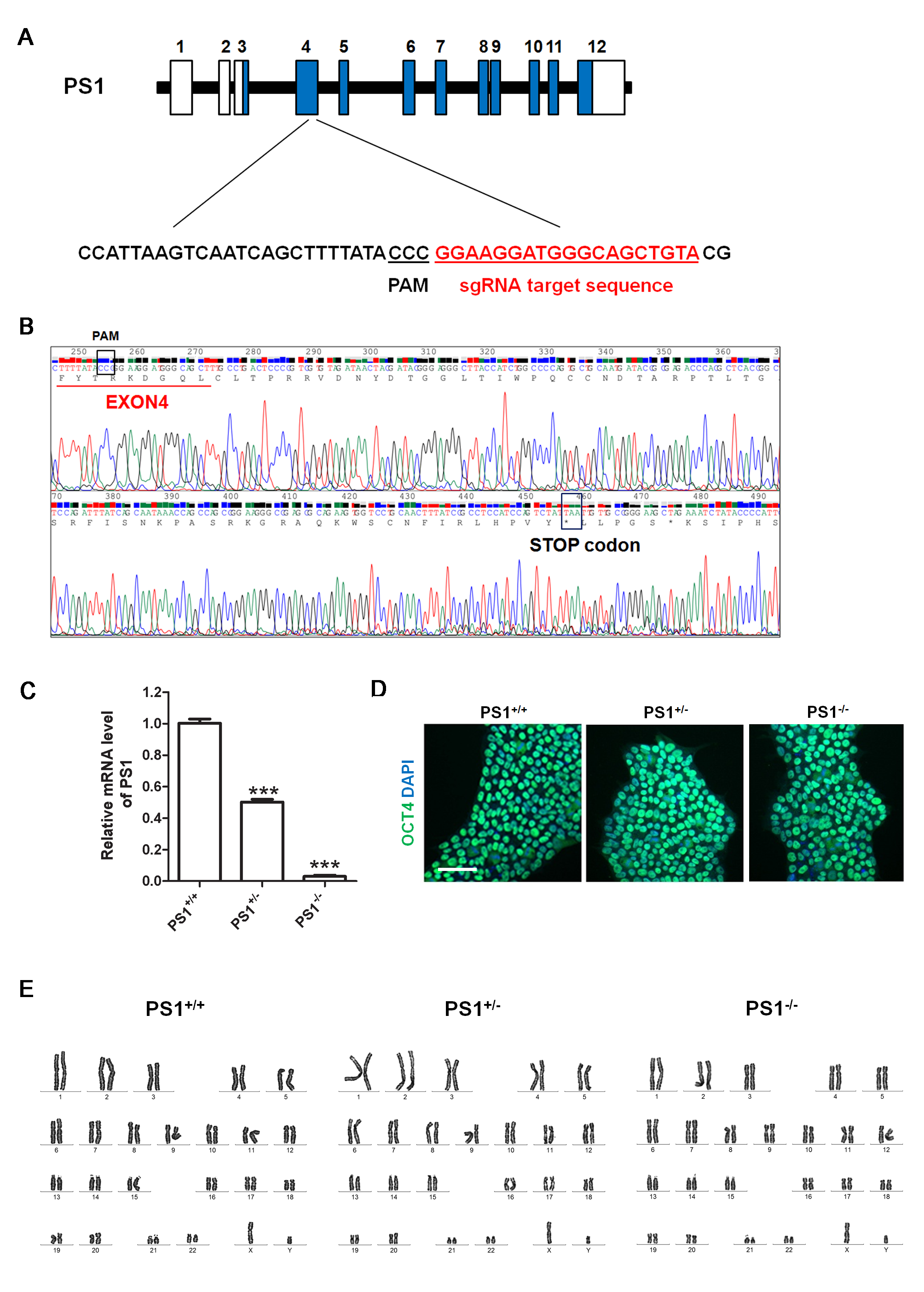

Supplement: Supplementary file 2 — Supplementary Figure 1 [file 41419_2018_945_MOESM2_ESM.tif]

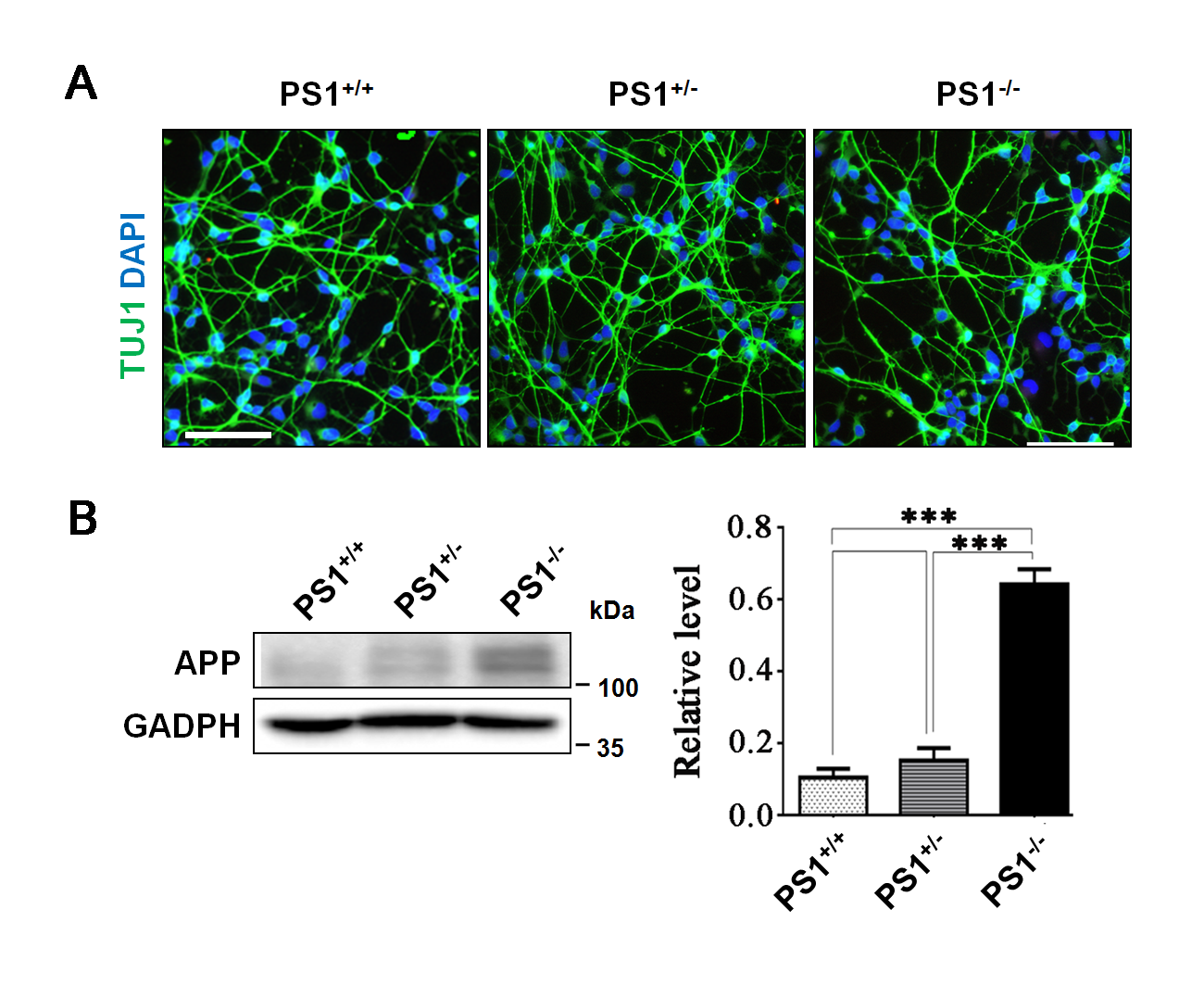

Supplement: Supplementary file 3 — Supplementary Figure 2 [file 41419_2018_945_MOESM3_ESM.tif]

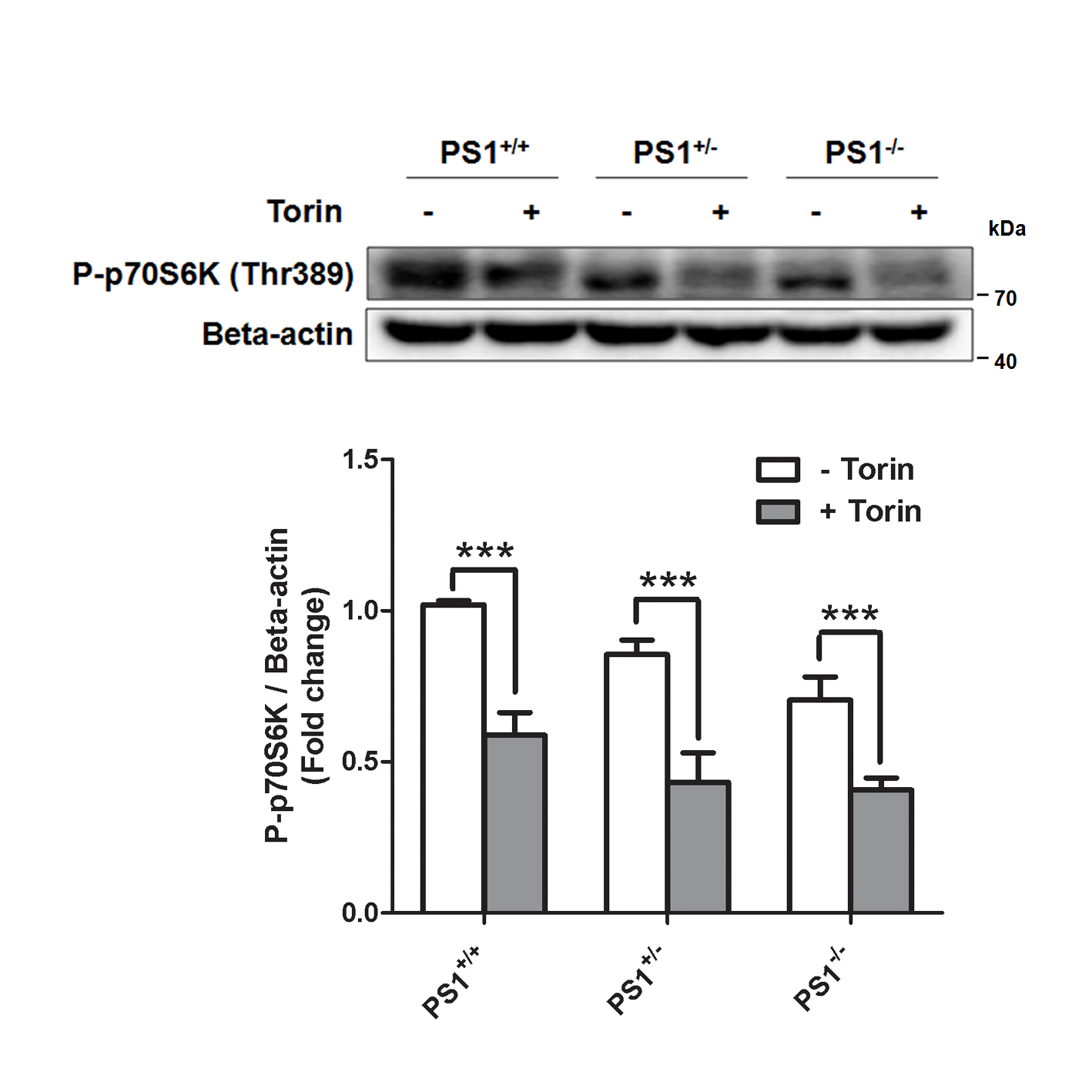

Supplement: Supplementary file 4 — Supplementary Figure 3 [file 41419_2018_945_MOESM4_ESM.tif]

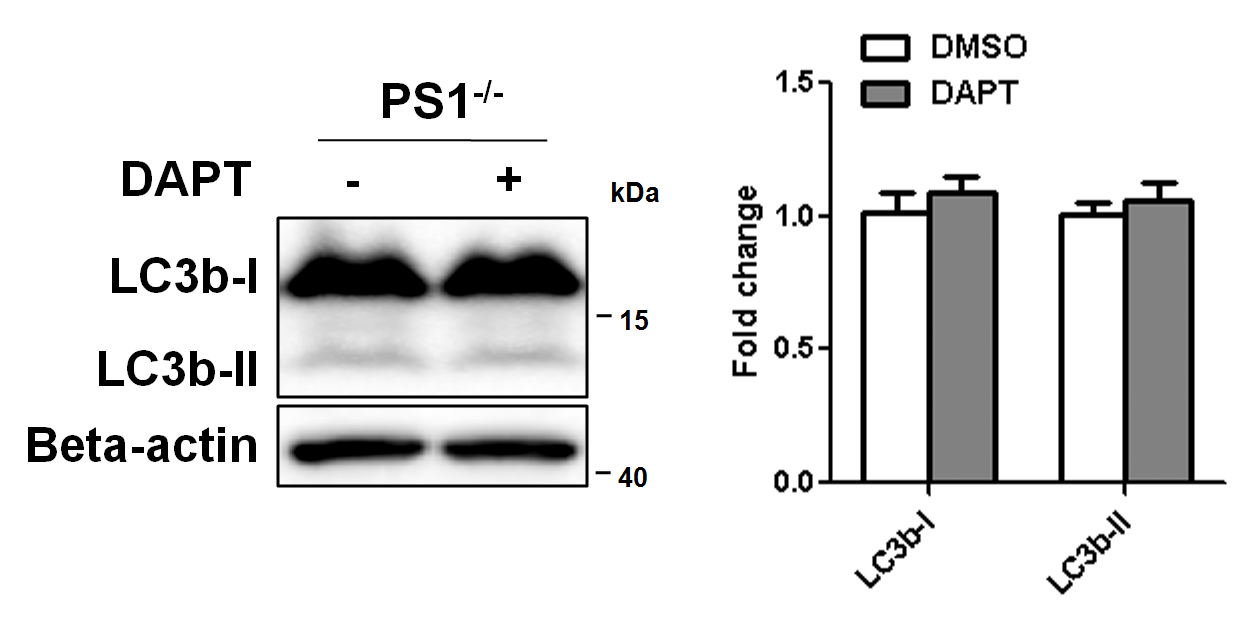

Supplement: Supplementary file 5 — Supplementary Figure 4 [file 41419_2018_945_MOESM5_ESM.tif]

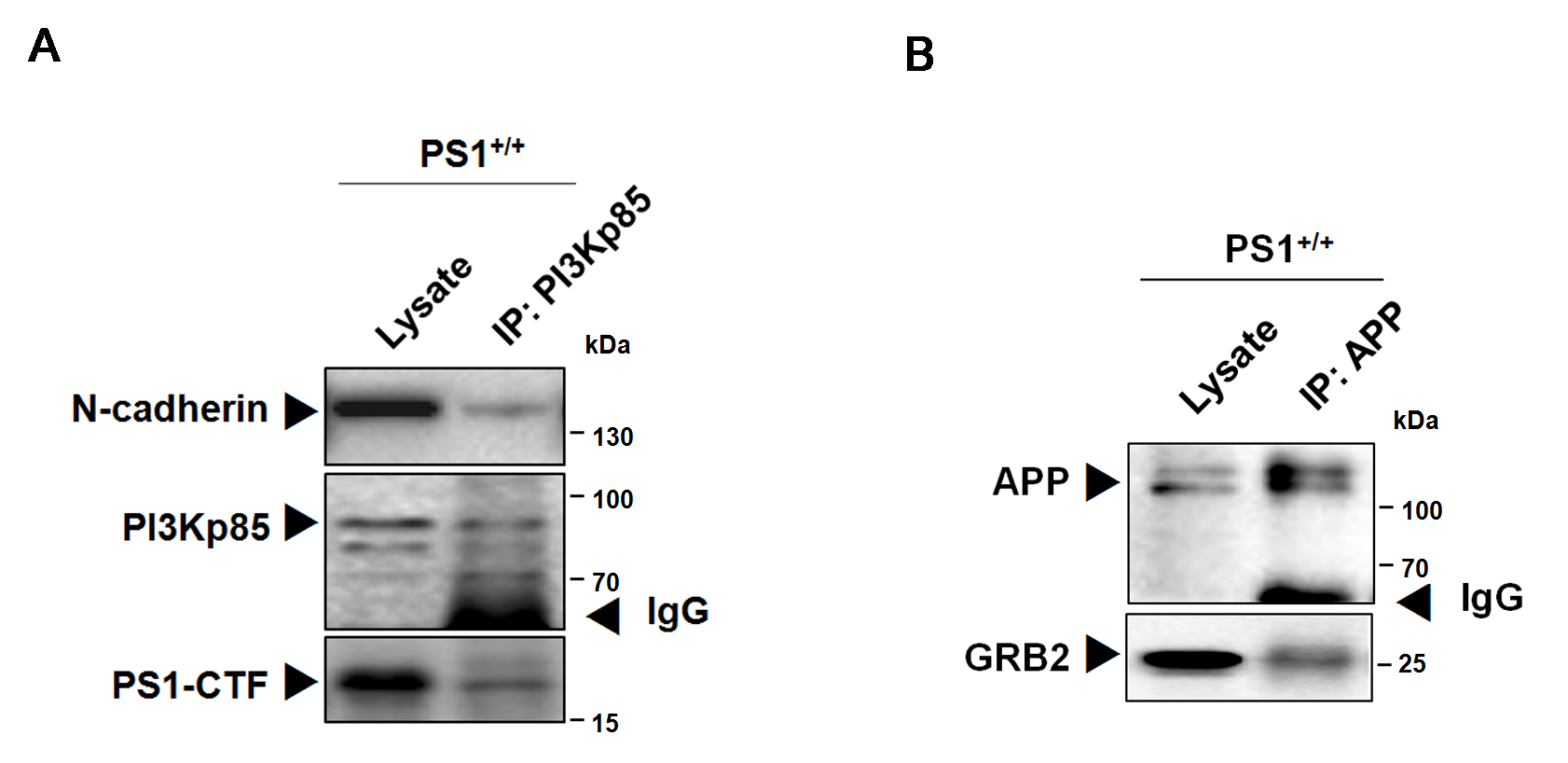

Supplement: Supplementary file 6 — Supplementary Figure 5 [file 41419_2018_945_MOESM6_ESM.tif]
